# Hydrological model-based streamflow reconstruction for Indian sub-continental river basins, 1951–2021

**DOI:** 10.1038/s41597-023-02618-w

**Published:** 2023-10-18

**Authors:** Dipesh Singh Chuphal, Vimal Mishra

**Affiliations:** 1 Civil Engineering, Indian Institute of Technology (IIT) Gandhinagar, Gandhinagar, India; 2 Earth Sciences, Indian Institute of Technology (IIT) Gandhinagar, Gandhinagar, India

**Keywords:** Hydrology, Hydrology

## Abstract

Streamflow is a vital component of the global water cycle. Long-term streamflow observations are required for water resources planning and management, hydroclimatic extremes analysis, and ecological assessment. However, long-term streamflow observations for the Indian-Subcontinental (ISC) river basins are lacking. Using meteorological observations, state-of-the-art hydrological model, and river routing model, we developed hydrological model-simulated monthly streamflow from 1951–2021 for the ISC river basins. We used high-resolution vector-based routing model (mizuRoute) to generate streamflow at 9579 stream reaches in the sub-continental river basins. The model-simulated streamflow showed good performance against the observed flow with coefficient of determination (R^2^) and Nash-Sutcliffe efficiency (NSE) above 0.70 for more than 60% of the gauge stations. The dataset was used to examine the variability in low, average, and high flow across the streams. Long-term changes in streamflow showed a significant decline in flow in the Ganga basin while an increase in the semi-arid western India and Indus basin. Long-term streamflow can be used for planning water management and climate change adaptation in the Indian sub-continent.

## Background & Summary

Climate change has altered the water cycle by changing the frequency and magnitude of the hydrological variables^1,2^. Understanding the changes in the water cycle is crucial for mapping the variability in the hydrological variables (e.g. precipitation, evapotranspiration, and streamflow). Streamflow is a vital component of the hydrological cycle, which provides an integrated response of a catchment to meteorological and other basin characteristics. Streamflow serves as a key input for water resources management and hydropower projects. In addition, flow in the rivers is critical to support the human activities and ecosystems that depend on river systems^3^. Long-term streamflow assessment is essential to examine streamflow variability, climate change impacts, and environmental protection^4–6^. Streamflow observations at gauge are the most reliable; however, they are limited in spatial and temporal coverage^7,8^. In addition, streamflow observations are not available for the three major transboundary basins (e.g. Ganga, Indus, and Brahmaputra) as they are classified. While streamflow observations are limited, accurate streamflow estimation using statistical and hydrological modelling remains a challenge for the water resources community.

Streamflow in India is monitored by the Central Water Commission (CWC). Despite having about 1500 streamflow monitoring stations in different river basins of India^9^, a number of streams across India remain unmonitored. Moreover, actively working monitoring stations with consistent data records do not cover smaller and remote streams. For instance, a majority of the streamflow monitoring sites provide 30–40 years of data, which is publicly available only for the peninsular river basins. To address this challenge, there is a need for solutions to offset the inconsistency and spatiotemporal limitations in streamflow measurements^10^. Hydrological modelling can supplement limited streamflow data in data-scarce ungauged regions^11–13^. However, the lack of high-quality input datasets, model parameterisation, and computational limits at high resolution may pose challenges for hydrological modelling^14–16^.

**Fig. 1:**
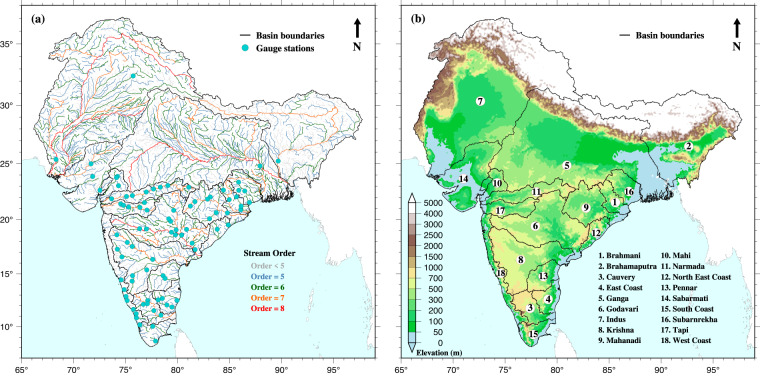
Map of the Indian subcontinent river basins showing the location of selected streamflow gauging station (cyan circles) and selected river segments. River segments of different stream order are represented in different colour. The thick black lines enclose the major ISC river basins. The shaded colour in the background in the right panel represents the elevation of the that area in meters from the mean sea level.

Global river routing models have undergone significant advancements in recent years, with improvements in routing methods, development of finer-scale river networks, and incorporation of anthropogenic water withdrawal^17–21^. Vector-based river routing models are preferred over grid-based models for their ability to provide a more realistic representation of river channels^22–26^. The vector-based routing models use vector river networks derived from high-resolution digital elevation models (DEMs). The finer-scale streamlines and other hydrological features are better represented in a vector-based river network than traditional grid-based network^24^. Yamazaki *et al*.^24^ reported that the vector-based river network utilised in CaMa-Flood was 60% more efficient than the grid-based river network in simulating observed streamflow. We used a vector-based mizuRoute model to simulate observed streamflow across 9579 stream reaches (Fig. 1a) in eighteen sub-continental river basins (Fig. 1b). We considered the stream reaches of all stream orders (levels 1–8). The highest stream order for sub-continental river basins (level 8) is for Ganga, Indus, and Godavari river basins (Fig. 1).

## Methods

### Workflow

We developed model-simulated monthly streamflow for the sub-continental river basins using observed meteorological forcing (precipitation, maximum and minimum temperatures) and land surface hydrological and river routing models for the 1951–2021 period. We obtained the daily observed meteorological forcing at 0.25° from the India Meteorological Department (IMD) and Sheffield *et al*.^27^ database, which was used to drive the H08 land surface model^28^. The H08 model simulations were conducted at 0.25° resolution. The runoff and baseflow produced by the H08 model were then used as input to the vector-based river routing model (mizuRoute) to simulate streamflow for each stream of the river network. The simulated streamflow was compared against the observed streamflow at the gauge stations where streamflow observations are available. We performed a manual calibration of the H08 model by adjusting the four parameters^29,30^ (soil depth, tau, gamma, bulk coefficient) to ensure reasonable agreement between the simulated and observed streamflow. We assessed the model’s performance using the Nash-Sutcliffe Efficiency^31^ (NSE) and coefficient of determination (R^2^) for monthly streamflow.

### Observed datasets

We obtained daily gridded precipitation data at 0.25° spatial resolution for the Indian region from IMD, covering the 1951–2021 period. The precipitation data was developed using daily measurements collected by a well-distributed network of rain gauges located across India. The gridded precipitation captures the spatial heterogeneity of rainfall across the region^32^. Similarly, daily maximum and minimum temperatures for the Indian region, spanning the period from 1951–2021, were obtained from IMD^33^ at a spatial resolution of 1°. The temperature data was developed using improved Shepard’s angular distance weighting algorithm^34^. Additionally, to ensure consistency with the gridded precipitation data, daily temperature data from IMD was bi-linearly interpolated to 0.25° using the Synergy Mapping algorithm^35^ considering the lapse rate. The high-resolution precipitation and temperature data have been used for streamflow simulations that are compared against observed streamflow^36–38^. Since IMD’s observational network covers only the Indian mainland, the meteorological data for the region outside India was obtained from Sheffield *et al*.^27^ database, which is available at 0.25° spatial and daily temporal resolutions. The Sheffield *et al*.^27^ precipitation and temperature agree reasonably well with the gridded observations from IMD for the Indian region and are consistent in terms of inter-annual variability and bias^39,40^. We compared Sheffield *et al*.^27^ and bias-corrected ERA5 (WFDE5)^41^ precipitation against IMD for the Indian region and observed similar performance of both datasets (Figure S1). However, the long-term availability of Sheffield *et al*.^27^ data (1901 onwards) made it suitable for this study.

The long-term observed monthly streamflow for the selected gauge stations was obtained from Central Water Commission (CWC) to evaluate the hydrological model’s performance. The gauge stations were chosen primarily based on data availability and the influence of the upstream reservoirs^40^. Moreover, the observed streamflow for the three transboundary rivers (Brahmaputra, Ganga, and Indus) was obtained from the Centre for Sustainability and the Global Environment (SAGE) database. The availability of publicly accessible streamflow for transboundary rivers was limited, and the number of gauge stations available was lesser than the other river basins mainly located in peninsular India. The performance of hydrological models in the sub-continental river basins (especially for the transboundary basins) has also been carefully evaluated for satellite-based soil moisture and evapotranspiration^42^. This is particularly important to ensure that the water budget in the transboundary river basins is well simulated by the model in the absence of streamflow observations.

### Hydrological and routing models

The H08 is a physically based large-scale water resources model, which generates the hydrological response in terms of daily runoff by employing the water and energy balance^28,43^. The H08 model is a single soil layer model that uses the bucket model approach for surface runoff estimation and the leaky bucket technique for subsurface runoff calculations^44^. The total runoff is divided into direct runoff and groundwater recharge, which is governed by the geology, relief, soil texture, and glacier indexes of the grid cell^45^. The H08 model uses the soil parameters from the Harmonized World Soil Database (HWSD) and land use data from the ISIMIP3a database^46^.

The mizuRoute is a vector-based river network routing tool developed by Mizukami *et al*.^25^ The vector-based river network and their corresponding hydrological response units (HRUs) input in the mizuRoute were taken from Hydrologic Derivatives for Modeling and Analysis (HDMA) database^47^. The vector layer (HRUs and streams) was derived from the 3 arc-seconds digital elevation model (DEM), comprising 9579 HRUs and streams distributed across the 18 major sub-continental river basins. The routing process involves the mapping of the gridded runoff to the river network HRUs by using the weightage-area runoff approach. The next steps involve hillslope routing, where a gamma-distribution-based unit-hydrograph is used to simulate the water flow on hillslopes, followed by river channel routing, which utilizes the kinematic wave tracking scheme to simulate water flow through the river channels.

### Reconstruction of streamflow

We integrated the H08 hydrological model and the mizuRoute routing model to develop long-term streamflow. We forced the H08 model with the observed meteorological data available at 0.25° spatial resolution. The simulations were carried out from 1951–2021 after stabilizing the initial spinup. The daily gridded runoff from the H08 model was routed along the vector-based river network by mizuRoute to produce spatially distributed discharge. Our hydrological modelling framework does not incorporate the role of human activities (i.e., irrigation, groundwater pumping, and reservoir storage) on streamflow. Human activities can considerably influence streamflow magnitude and variability^48^. However, the observational datasets to estimate the role of human activities on streamflow variability are unavailable at appropriate temporal and spatial scales. We manually calibrated the combined modelling system for each basin by adjusting four input variables of the H08 model. The four variables include soil depth (meters), tau (days), gamma (dimensionless), and bulk transfer coefficient (parameter for estimating potential evaporation). The sensitivity analysis demonstrated that these four critical parameters significantly influence runoff generation^49^. The calibration parameters of H08 are discussed in detail in Dangar & Mishra^29^. We used different combinations of these parameters by systematically adjusting them within the defined range of each parameter (Table S1). The parameters employed for the 18 river basins within the Indian Subcontinent are available in supplementary information (Table S2). Since the model calibration is performed against the observed streamflow instead of naturalized streamflow, the calibrated parameters can account for the influence of human activities^50^. The model simulated streamflow was obtained at a daily time scale and for each stream considered in the river network. We estimated the mean monthly streamflow from the daily discharge of the mizuRoute for each stream.

We estimated standardised precipitation index (SPI) and standardised streamflow index (SSI) for the assessment of drought conditions. SPI and SSI are dimensionless drought indices that are used to identify anomalous dry and wet periods based on precipitation and streamflow data^51–53^. We used the SPI and SSI values to represent the relationship between precipitation deficits and their impacts on streamflow. We employed the parametric method for estimating the SPI and SSI by fitting the gamma distribution to the data. The data is transformed into a standard normal distribution using the cumulative distribution function of the assumed gamma distribution. The positive values (greater than 0.5) of SPI and SSI indicate wetter conditions, while negative values (less than −0.5) indicate drier conditions. SPI and SSI between −0.5 and 0.5 indicate normal condition.

## Data Records

Monthly streamflow dataset (m^3^/s) for the period 1951 to 2021 are available from Zenodo repository^54^ for all the selected streams in the sub-continental river basins. The mean annual and monsoon streamflow (m^3^/s), mean low and high flow (m^3^/s), and coefficient of variation (CV) in annual and monsoon streamflow were estimated for all the streams. Additionally, the SSI for the top four dry and wet months during the 1951–2021 period was estimated. Moreover, the data repository includes a list of stream reaches that exhibit a statistically significant trend in streamflow between 1951 and 2021. We assigned a unique identification (ID) to each stream segment of the sub-continental river basins. The segment ID (seg_id) corresponding to a stream can be found in the attribute table of the stream’s shapefile (India_streams) available in the above directory. A readme file available at the above link provides detailed information on the format of the data.

## Technical Validation

**Fig. 2:**
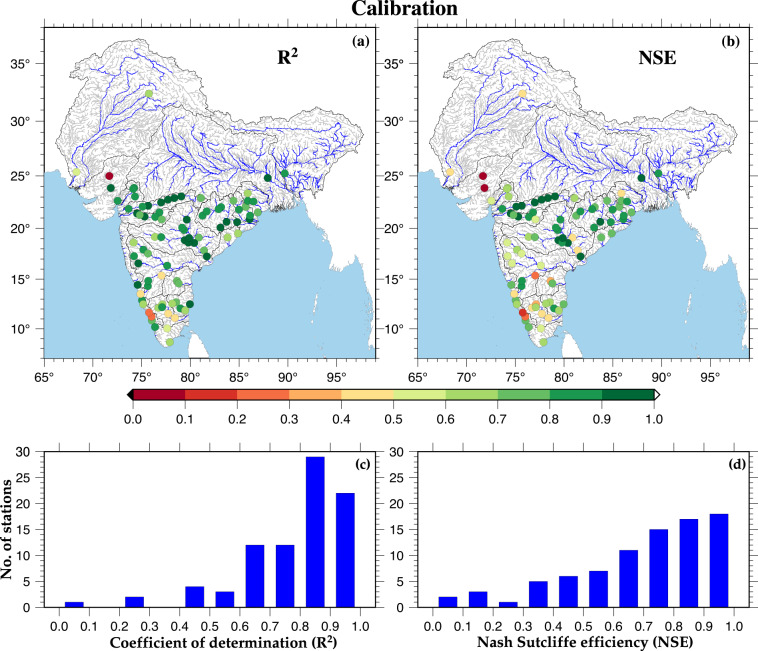
Calibration of model against monthly streamflow. (**a,b**) Spatial distribution of R^2^ and NSE value between observed and simulated monthly streamflow for selected gauge stations. (**c,d**) Distribution of stations based on R^2^ and NSE values during model calibration.

**Fig. 3:**
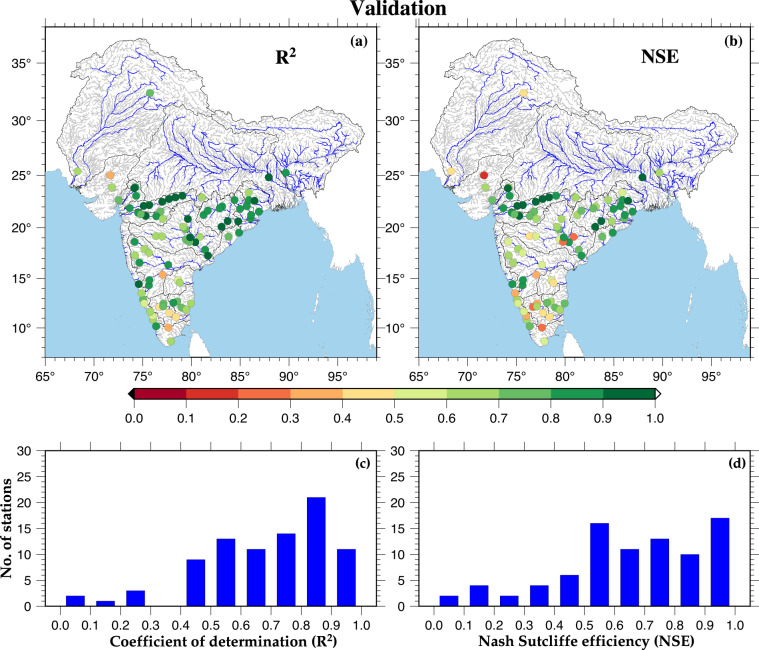
Validation of model against monthly streamflow. (**a,b**) Spatial distribution of R^2^ and NSE value between observed and simulated monthly streamflow for selected gauge stations. (**c,d**) Distribution of stations based on R^2^ and NSE values during model validation.

We compared the observed and simulated monthly streamflow for 85 streamflow gauge stations across India. We utilized approximately half of the period for which observed streamflow is available for the model calibration and the other half for the evaluation of the hydrological model. Thus, we considered about 10 to 15 years for the calibration and validation to ensure that the model captures temporal patterns and seasonality in streamflow. We evaluated the performance of the H08 model by estimating the Coefficient of Determination (R^2^) and Nash-Sutcliffe Efficiency^31^ (NSE) at all gauge stations. R^2^ indicates how well the model explains the variation in the observed streamflow, while NSE compares the model-simulated streamflow to the observed flow, taking into account the variability in streamflow. The H08 model performed well (satisfactory R^2^ and NSE) in simulating streamflow for the sub-continental river basins (Figs. 2, 3). The majority of the stations show NSE and R^2^ more than 0.60 for both calibration and evaluation (Table S3). The average R^2^ and NSE values for the calibration were 0.78 and 0.69, while for evaluation were 0.76 and 0.67, respectively. More than 60% of the selected stations show R^2^ and NSE greater than 0.70 during the model calibration and validation. However, a few stations show NSE and R^2^ less than 0.50. The H08 model exhibited relatively good performance (NSE and R^2^ > 0.7) at gauge stations in the Narmada, Mahi, Tapi, Mahanadi, Ganga, and Brahmaputra river basins. In contrast, the model performance is relatively weaker (NSE and R^2^ < 0.5) at locations in Cauvery, Sabarmati, and Indus river basins, which can be attributed to the quality of meteorological and streamflow observations or other input parameters related to soil and vegetation^30,37^. The number of selected gauge stations in Indus, Ganga, and Brahmaputra river basins was less than others due to confidentiality in the publicly available streamflow data of the transboundary river basins.

We also evaluated the model’s performance (R^2^ and NSE) for the daily streamflow at 80 streamflow gauge stations across India (Figure S2, Table S4). Since we have calibrated the model for the monthly streamflow, the model’s performance was reduced at a few stations while evaluating for the daily streamflow. The average R^2^ and NSE during calibration and validation of the model for daily streamflow was satisfactory (more than 0.50). We next evaluated the performance of trends in simulated mean monsoon streamflow against observed streamflow during the period 1981–2010 for six streamflow gauge stations (that had long-term observations without considerable missing data) across India (Figure S3). We selected a 30-year common period (1981–2010) for each station based on the observed streamflow availability. Both the observed and simulated flow are consistent for the long-term trends for 1981–2010 period. The simulated streamflow effectively captures the interannual variability in the observed streamflow (Figure S3). The model performed well for most stations in different river basins, including the Narmada, Brahmani, and Mahi river basins (Figure S3a–c).

**Fig. 4:**
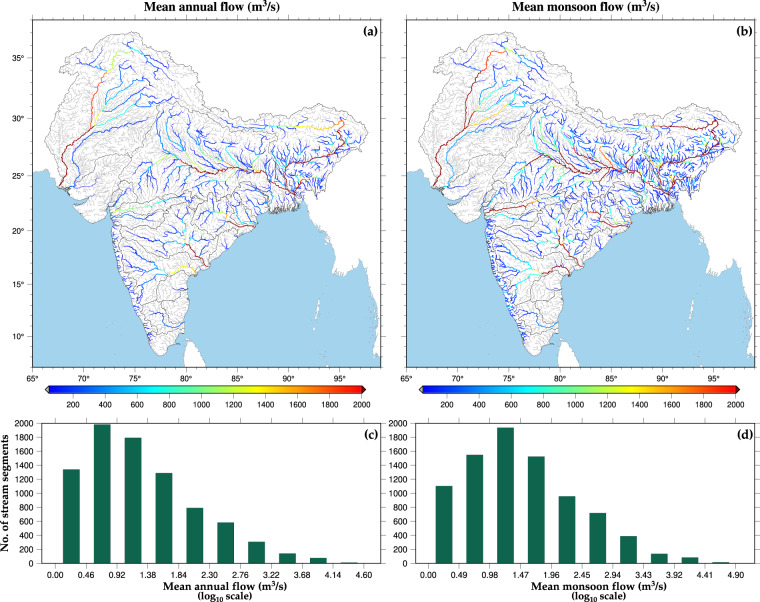
Long-term historical (1951–2021) mean flow in Indian subcontinent river basins. (**a,b**) Mean annual and monsoon historical flow (m^3^/s) of selected ISC river segments. Grey colour represents the river segments having flow less than 50 m^3^/s. (**c,d**) Distribution of streams based on mean annual and monsoon flow. The x-axis is made on a logarithmic scale (log_10_ scale).

We utilised the hydrological model simulated monthly streamflow data to estimate the mean annual and mean monsoon flow in the stream reaches of sub-continental river basins during 1951–2021 (Fig. 4). The stream reaches with a mean flow of less than 50 m^3^/s were depicted in grey (Fig. 4a,b). The rivers Indus, Ganga, Brahmaputra, Mahanadi, and Godavari have higher mean annual and monsoon flow (greater than 2000 m^3^/s). Additionally, Narmada and Krishna exhibit a mean monsoon season flow greater than 2000 m^3^/s, while their mean annual flow is less than 1000 m^3^/s. We use log transformation (base 10) to reduce the variability in mean flow of the small and large streams. The log-transformed mean annual flow in sub-continental rivers varies between 0 and 4.60 (Fig. 4c). At the same time, the mean monsoon flow varies between 0.0 and 4.90 (Fig. 4d). Most of the stream reaches have a log-transformed mean annual and mean monsoon flow less than 1.84 and 1.96, respectively. The coefficient of variation (CV) in mean annual and mean monsoon flow for the stream reaches having flow of more than 50 m^3^/s ranges between 0 and 4 (Figure S4). The CV for annual flow is consistently higher than that of monsoon flow across all stream reaches. The rivers Indus, Ganga, and Brahmaputra have the minimum CV in streamflow due to the year-round contribution of glacier and snow melt water. Moreover, the CV for stream reaches in the Brahmaputra river basin is lower than that of other river basins, indicating that the Brahmaputra basin has the most consistent streamflow. The analysis can be used in decision and policy-making for rivers that have higher variability in streamflow availability over the year.

**Fig. 5:**
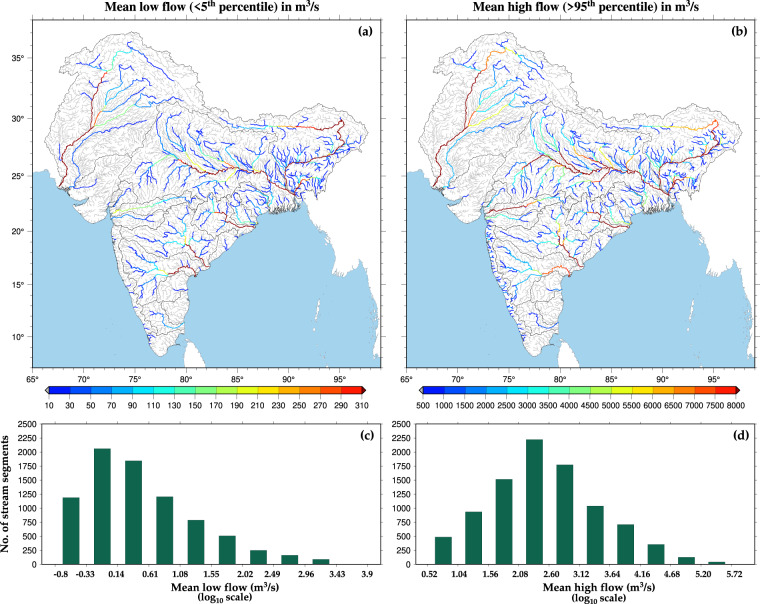
Long-term historical (1951–2021) mean low and high flow in ISC river basins. (**a**) Mean low flow (m^3^/s) of selected ISC river segments calculated as the average of streamflow values less than 5^th^ percentile of the time series (1951–2021). Grey colour represents the river segments having flow less than 10 m^3^/s. (**b**) Mean high flow (m^3^/s) of selected ISC river segments calculated as the average of streamflow values higher than 95^th^ percentile of the time series (1951–2021). Grey colour represents the river segments having flow less than 500 m^3^/s. (**c**) & (**d**) Distribution of streams based on mean low and high flow. The x-axis is made on a logarithmic scale (log_10_ scale).

We also estimated the mean low flow and high flow for the stream reaches of the sub-continental river basins (Fig. 5). We calculated the mean low flow and high flow by averaging the streamflow below the 5^th^ percentile and above the 95^th^ percentile thresholds of the time series (1951–2021), respectively. The mean low flow and high flow have similar spatial variation across all the streams but differ in absolute magnitude (Fig. 5a,b). The mean low flow transformed in the log scale ranges between −0.8 and 3.9 (Fig. 5c). In contrast, the mean high flow transformed in the log_10_ scale varies between 0.52 and 5.72 (Fig. 5d). Overall, the log-transformed flow value is less than 1.55 and 3.12 for mean low flow and mean high flow, respectively.

**Fig. 6:**
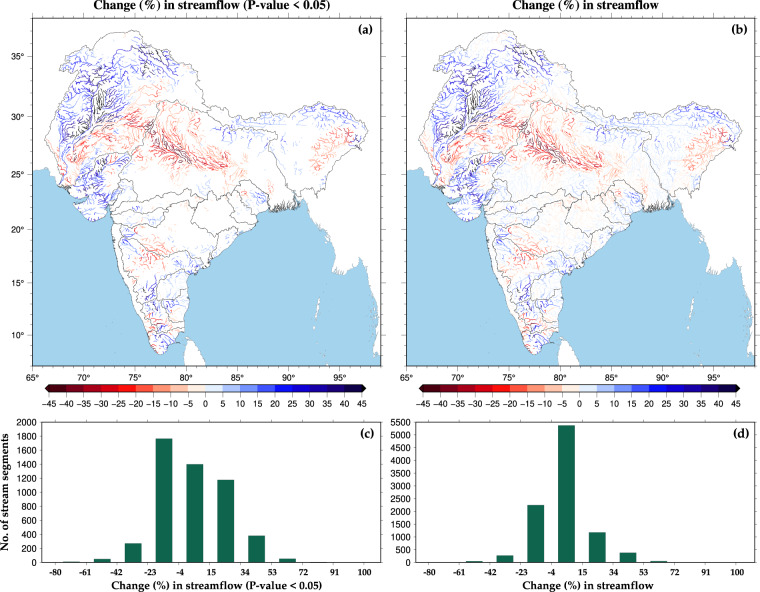
Percentage change in monthly flow between 1951 and 2021 for ISC river basins. (**a,b**) Percentage flow change for streams showing statistically significant trend based on Mann Kendall trend analysis test and for overall river segments. (**c,d**) Distribution of river segments based on percentage flow change for statistically significant and overall streams.

Using the simulated streamflow, we evaluated the stream reaches that have a statistically significant trend in streamflow during 1951–2021 (Fig. 6a). We used the modified Mann-Kendall trend analysis test to evaluate the streams having statistically significant trends in streamflow^55,56^. We find that more than 5000 stream reaches show a statistically significant trend in streamflow (Fig. 6c). Stream reaches in the Mahanadi, Narmada, Godavari, and West Coast river basins do not exhibit a considerable trend in streamflow, while the Indus, Ganga, and Sabarmati river basins experienced substantial changes in streamflow (Fig. 6a). The percentage decrease in streamflow is the highest for stream reaches in the Ganga river basins. In contrast, the percentage increase in streamflow is most prominent for stream reaches in the Sabarmati and Indus river basins. We estimated changes (%) in streamflow for all the stream reaches, including both significant and insignificant streamflow trends (Fig. 6b), and their distribution based on percentage flow change (Fig. 6d). Streamflow change for most of the stream reaches (>5000 streams) varies between −4% to 15% (Fig. 6d). However, on average, stream reaches in the sub-continental river basins experienced an overall increase in streamflow during recent decades (1951–2021).

We next estimated SSI for each stream reach for the top four dry and wet monsoon months between 1951 and 2021 (Figures S5, S6). We determined the top four driest and wettest months based on SPI calculated using monthly average precipitation data for the Indian Subcontinent. The four driest monsoon months for India were July 2002, June 2009, July 1972, and June 2014. SSI value ranges between −4.0 to −0.5 for the majority of the stream reaches during all four months (Figure S5e–h). Similarly, the four wettest monsoon months for India were July 1988, August 2020, September 2019, and September 1983. The SSI value for the above four months varies between −0.5 to 6.0 (Figure S6e,–h).

**Fig. 7:**
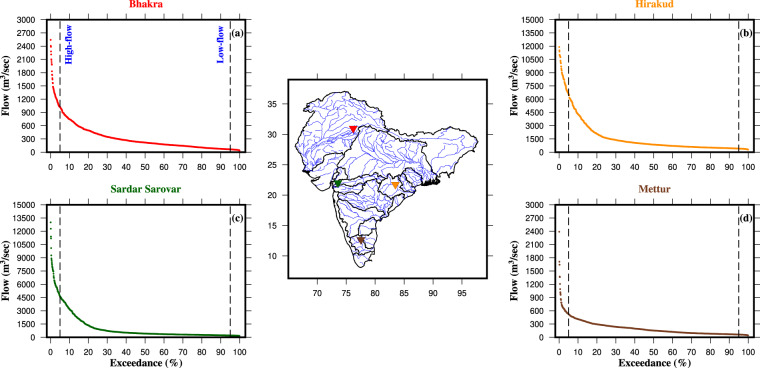
Flow duration curves at the upstream of the hydropower projects in India. (**a,b**), (**c,d**) Monthly flow duration curve for the Bhakra, Hirakud, Sardar Sarovar, and Mettur dams, respectively. The black dashed lines represent the threshold of high-flow (<5%) and low-flow (>95%) events. Map in the central panel shows the location of the selected hydropower dams across India.

The model-simulated streamflow can provide valuable insights for optimizing the capacity and functionality of dams and reservoirs. We examined the monthly flow duration curves at upstream of four prominent hydropower dams in India: Bhakra, Hirakud, Sardar Sarovar, and Mettur (Fig. 7). We strategically selected these dams from different regions of India, representing the northern, eastern, western, and southern parts of the country, respectively. Our analysis of the monthly flow duration curves for the selected dams revealed intriguing findings. The monthly flow for Bhakra, Sardar Sarovar, and Mettur was less than 400 m^3^/s for more than 50% of the time. In contrast, the Hirakud dam exhibited a 50% flow rate of around 750 m^3^/s. Furthermore, the high-flow events (flow available for less than 5% of the time) ranges between 500 m^3^/s to 2500 m^3^/s for Mettur and 1000 m^3^/s to 2700 m^3^/s for Bhakra (Fig. 7a,d). In the case of Hirakud, high-flow varies between 6000 m^3^/s and 12000 m^3^/s (Fig. 7b). Notably, Sardar Sarovar exhibited the widest range of high-flow values (upto 13500 m3/s), while it demonstrated the sharp dip at the low-flow end of the flow duration curve (Fig. 7c), which can be attributed to increased atmospheric demands at higher temperature during dry months. Flow duration curves for Sardar Sarovar and Mettur demonstrated a steep slope at the high-flow end, indicating rapid runoff attributed to substantial rainfall events during the wet months. Conversely, the curve for Bhakra exhibited a more gradual slope, indicating a significant contribution of the snowmelt water (Fig. 7a). These dams play a critical role in India’s hydropower generation and water resource management, and the analysis of the monthly flow duration curves provides valuable insights into the hydrological characteristics of these dams. The simulated streamflow can enhance the understanding of flow patterns and water availability dynamics of these dams, aiding in informed decision-making for efficient hydropower generation and water resource management strategies.

## Usage Notes

Streamflow variability in the sub-continental river basins has increased during recent decades. Long-term consistent streamflow is vital for flood and drought monitoring, ecological assessment, and water resources management. We simulated streamflow using physically based hydrological models (H08-mizuRoute). We used high-resolution vector-based river routing model (mizuRoute) to simulate streamflow for more than 9500 stream reaches in the sub-continental river basins. The model simulated streamflow was evaluated against observed monthly streamflow at 85 stream gauge stations across India. The validation ensures that the data accurately represents the temporal variability and seasonality of streamflow. The long-term streamflow data can be used to assess the hydrologic alteration and its ecological impacts. The model simulated streamflow can also be used to identify stream reaches that are more susceptible to hydroclimatic extremes, such as floods and droughts, which can improve local flood and drought awareness and redefine regional policies.

While we have evaluated the simulated streamflow data against observed streamflow, there are limitations. For instance, we considered Sheffield *et al*.^27^ precipitation and temperature for the region outside of India. However, the data may have a bias in the mountainous regions due to the limited number of available meteorological stations. Moreover, the monthly temporal resolution of streamflow could potentially pose limitations for certain applications that require daily data, like capturing rapid changes in streamflow during floods. Furthermore, we did not consider reservoir operations and anthropogenic withdrawal of water from rivers while calibrating the model. Despite these limitations, the overall performance of the model simulated streamflow was satisfactory (average R^2^ and NSE > 0.67). The hydrological model simulated streamflow can also support climate change adaptation by assessing the historical changes in streamflow for the long-term sustainability of water resources. The data can facilitate environmental assessments, enabling the evaluation of the ecological health of rivers and their surrounding ecosystems. Additionally, the dataset can assist in the assessment of river-induced greenhouse gas contributions to the global carbon budget.

### Supplementary information

Supplementary Table S4

Supplementary Information

Supplementary Table S3

## Data Availability

Publicly available source codes and manuals for H08 and mizuRoute were downloaded from http://h08.nies.go.jp and https://github.com/ESCOMP/mizuRoute. Model parameters detail for each basin is available in the supplement information. Data processing and plotting were performed using MATLAB and Generic Mapping Tool 6 (GMT 6), whereas QGIS 3.22 was utilized for geospatial analysis. The MATLAB codes used for data processing can be accessed through the GitHub directory (https://github.com/DIPESHSINGHCHUPHAL/Streamflow-India).
