# Supplementary material for: Hydrological model-based streamflow reconstruction for Indian sub-continental river basins, 1951–2021
Source: Sci Data. 2023 Oct 18;10:717. doi: 10.1038/s41597-023-02618-w (PMC10584895; doi:10.1038/s41597-023-02618-w)
Supplement: Supplementary file 2 — Supplementary Information [file 41597_2023_2618_MOESM2_ESM.docx]

**Supplemental Information**

**Hydrological model-based streamflow reconstruction for Indian sub-continental river basins, 1951-2021**

Dipesh Singh Chuphal^1^ and Vimal Mishra^1,2^

1. Civil Engineering, Indian Institute of Technology (IIT) Gandhinagar

2. Earth Sciences, Indian Institute of Technology (IIT) Gandhinagar

Corresponding author: [vmishra@iitgn.ac.in](mailto:vmishra@iitgn.ac.in)

**Table of contents**

| **Figure/Table** | **Page No.** |
| --- | --- |
| Figure S1 | 2 |
| Figure S2 | 3 |
| Figure S3 | 4 |
| Figure S4 | 5 |
| Figure S5 | 6 |
| Figure S6 | 7 |
| Table S1 | 8 |
| Table S2 | 8 |


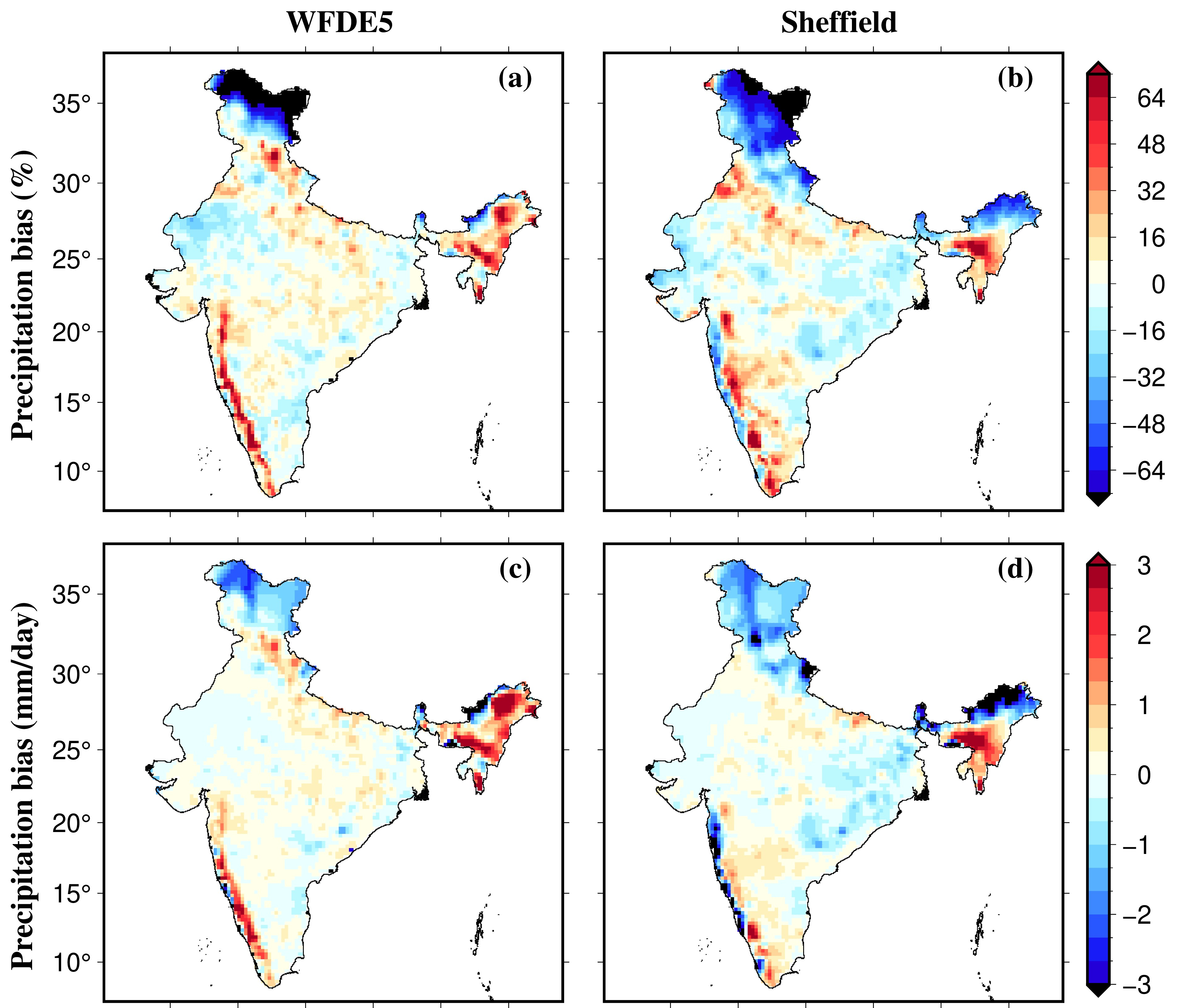


Figure S1: Bias in WFDE5 and Sheffield precipitation against reference IMD precipitation for the Indian region between 1981-2010.


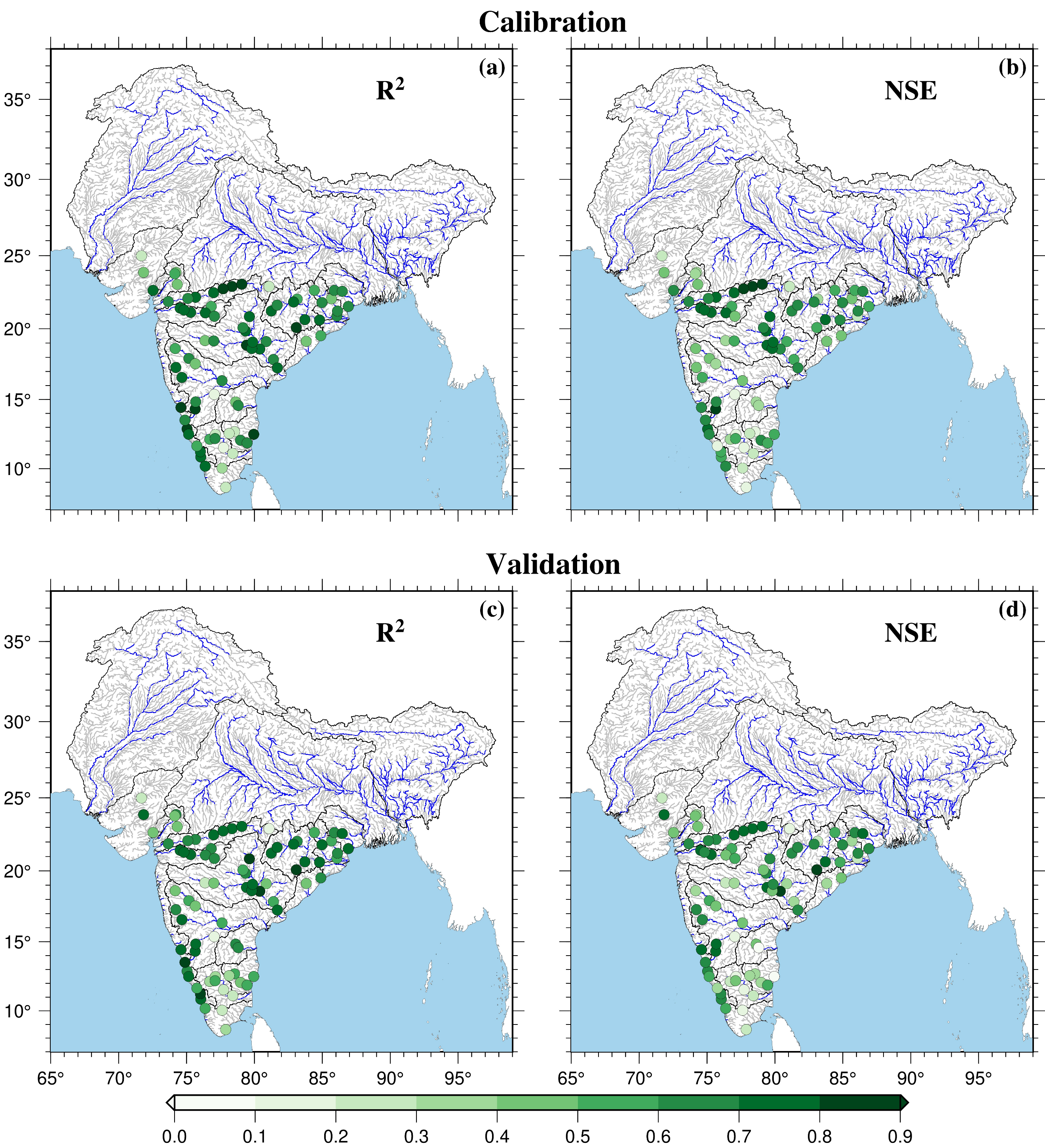


Figure S2: Model’s performance skills against daily streamflow. (**a**) & (**c**) Spatial distribution of R^2^ value between observed and simulated daily streamflow during calibration and validation for selected gauge stations. (**b**) & (**d**) Spatial distribution of NSE value between observed and simulated daily streamflow during calibration and validation for selected gauge stations.


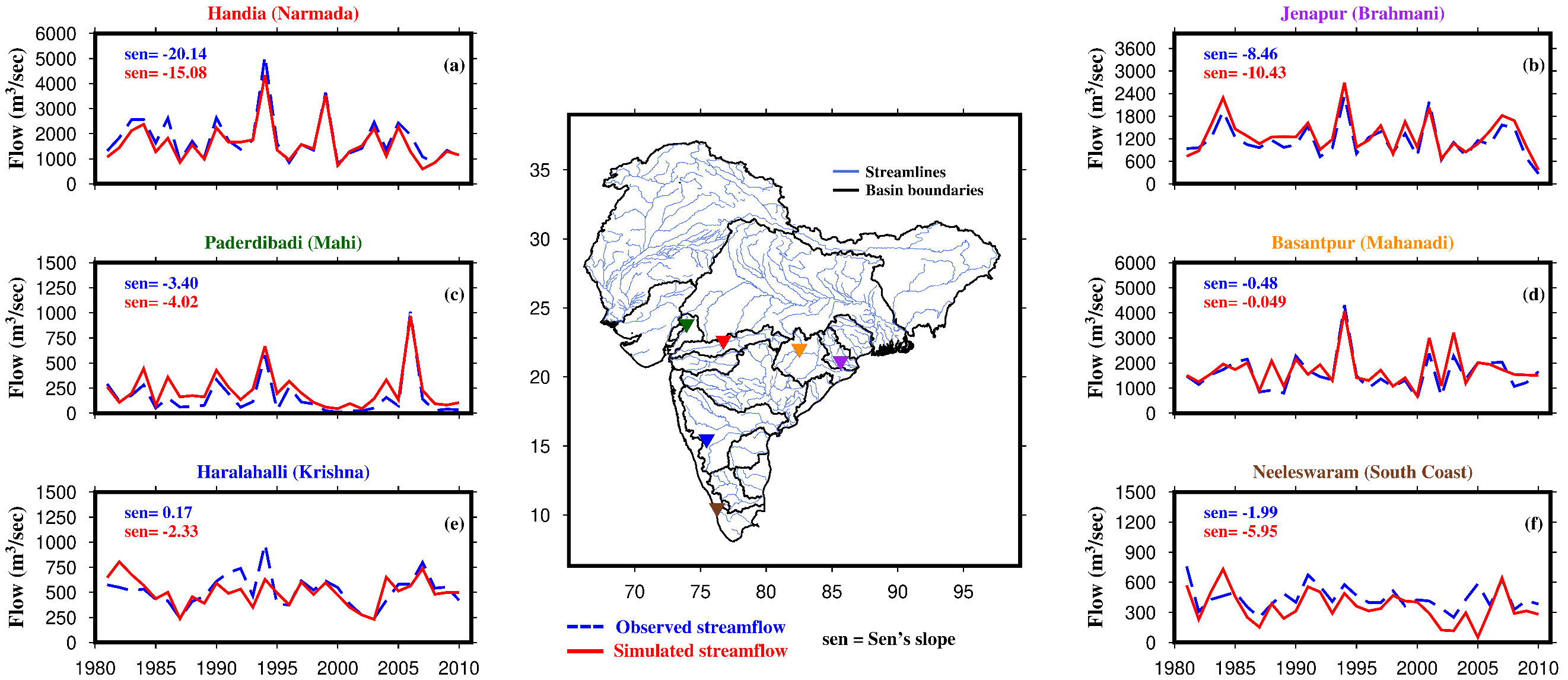


Figure S3: Comparison of trend between observed and simulated mean monsoon streamflow during the period 1981-2010 for gauge stations (**a**) Handia, (**b**) Jenapur, (**c**) Paderdibadi, (**d**) Basantpur, (**e**) Haralahalli, and (**f**) Neeleswaram. The observed mean monsoon flow (m^3^/s) is depicted using blue dashed lines, while the simulated mean monsoon flow (m^3^/s) is indicated by red lines. The central panel displays a map highlighting the geographical locations of the selected streamflow gauge stations across India. The Sen's slope values in each panel signify the trend gradient for both observed and simulated flows, computed using the Mann-Kendall test.

**
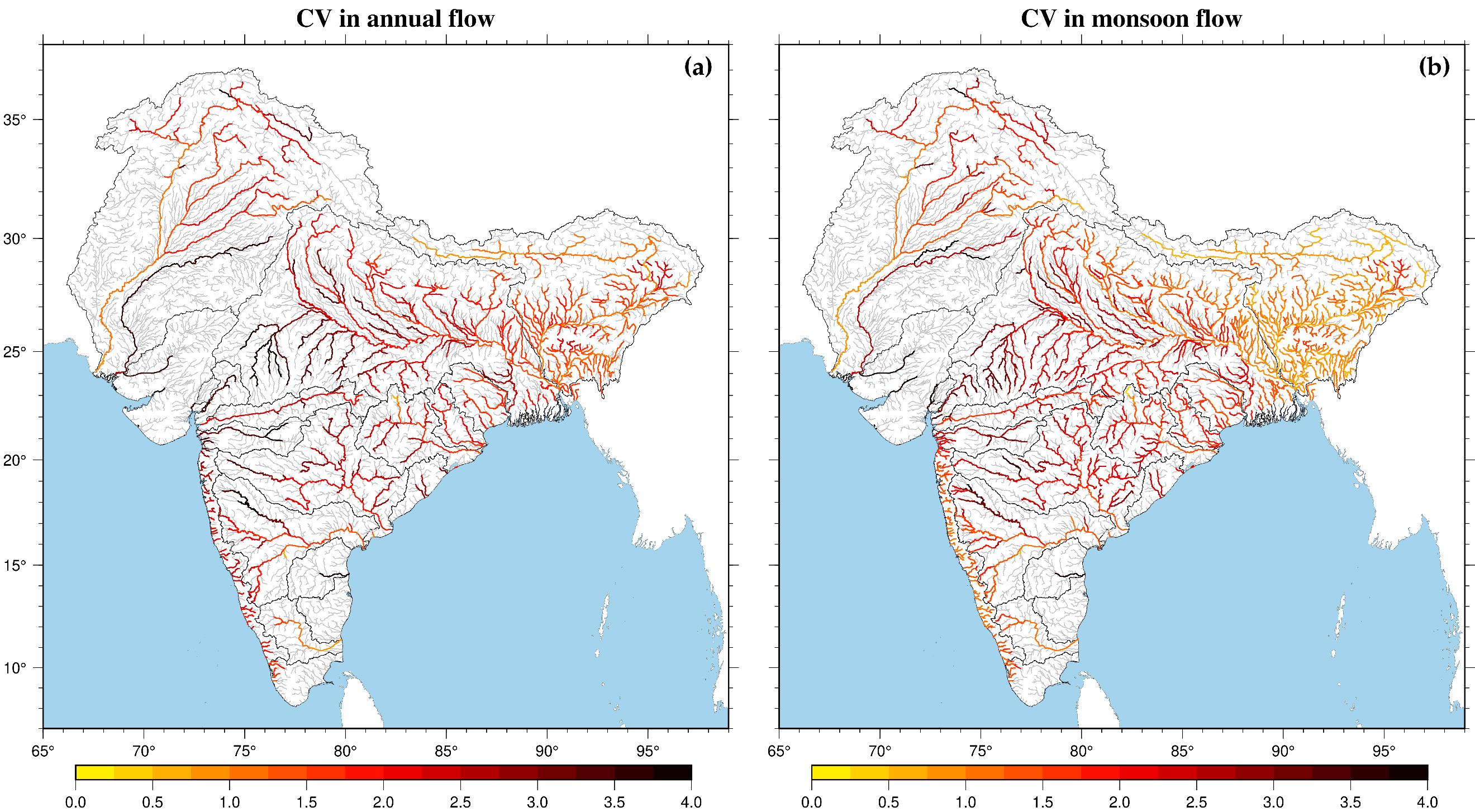
**

Figure S4: Coefficient of Variation (CV) in streamflow for selected river segments of ISC river basins. (**a**) & (**b**) CV in annual and monsoon flow between 1951 and 2021 respectively.

**
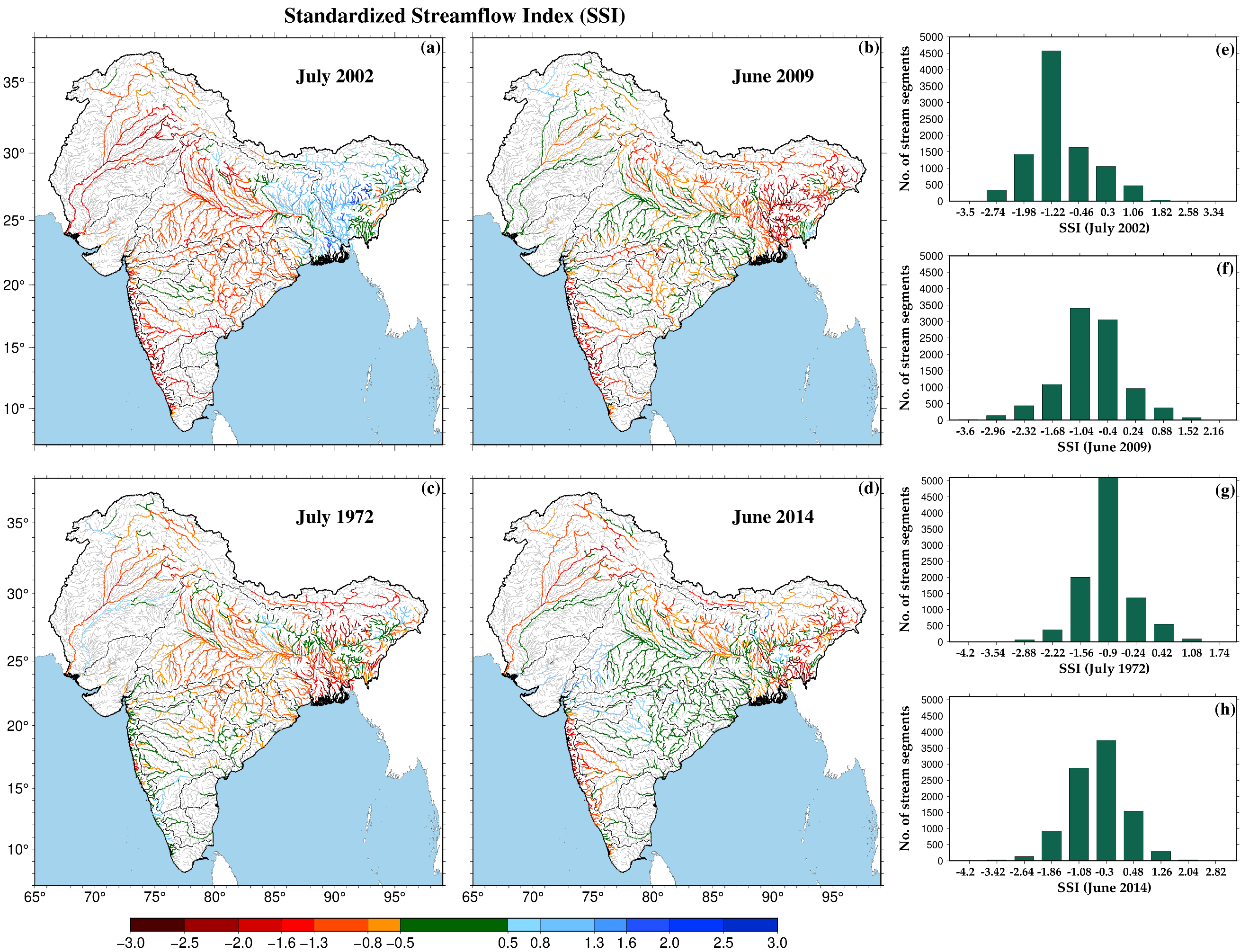
**

Figure S5: Standardized streamflow index (SSI) for ISC river segments for dry years. (**a**), (**b**), (**c**) & (**d**) Spatial distribution of SSI for top 4 dry months between 1951 to 2021 based on standardized precipitation index (SPI). SSI for the river segments having flow less than 50 m^3^/s is shown in grey for better visualization between major and minor river segments. (**e**), (**f**), (**g**) & (**h**) Distribution of streams based on SSI during July 2002, June 2009, July 1972 and June 2014, respectively.


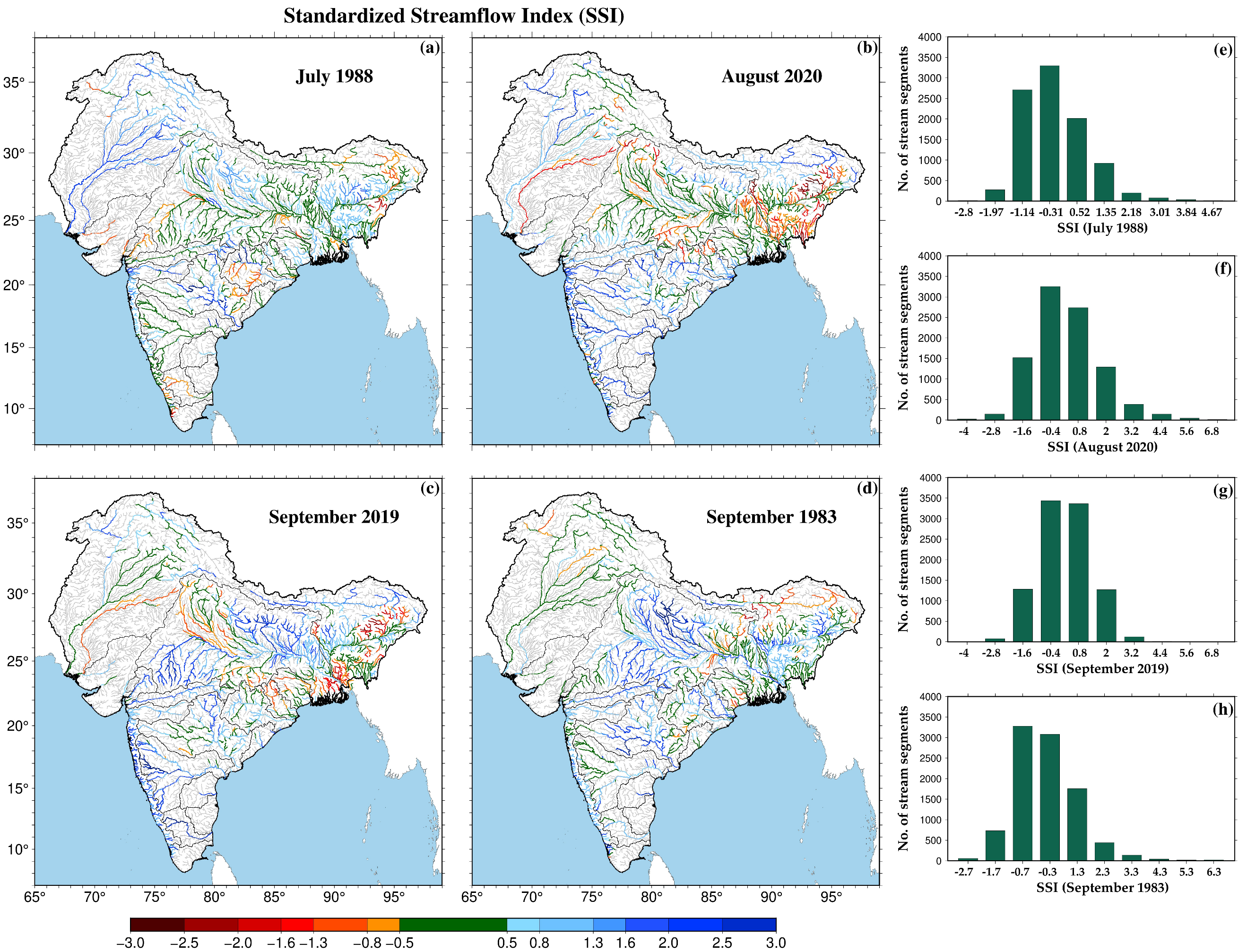


Figure S6: Standardized streamflow index (SSI) for ISC river segments for wet years. (**a**), (**b**), (**c**) & (**d**) Spatial distribution of SSI for top 4 wet months between 1951 to 2021 based on standardized precipitation index (SPI). SSI for the river segments having flow less than 50 m^3^/s is shown in grey for better visualization between major and minor river segments. (**e**), (**f**), (**g**) & (**h**) Distribution of streams based on SSI during July 1988, August 2020, September 2019 and September 1983, respectively.

Table S1: Range of critical parameters used in the evaluation of the H08 model.

| **Parameters** | **Range** | **Unit** | **Estimation** |
| --- | --- | --- | --- |
| Soil depth | 1-5 | Meter | Soil wetness |
| Bulk transfer coefficient | 0.001-0.01 | Dimensionless | Potential evaporation |
| Gamma | 1-4 | Dimensionless | Subsurface runoff |
| Tau | 30-300 | Days | Subsurface runoff |

Table S2: Critical parameters value corresponding to each basin used in the evaluation of the H08 model.

| **Basin Name** | **Bulk transfer coefficient** | **Soil depth** | **Tau** | **Gamma** |
| --- | --- | --- | --- | --- |
| Brahmani | 0.002 | 2.8 | 105 | 2.8 |
| Brahmaputra | 0.001 | 1.75 | 70 | 2.1 |
| Cauvery | 0.009 | 4.5 | 105 | 2.1 |
| East Coast | 0.009 | 2 | 300 | 2.8 |
| Ganga | 0.003 | 1 | 155 | 3.6 |
| Godavari | 0.003 | 2.8 | 105 | 2.8 |
| Indus | 0.001 | 1 | 155 | 1 |
| Krishna | 0.005 | 3.6 | 155 | 1.5 |
| Mahanadi | 0.002 | 2.8 | 105 | 2.8 |
| Mahi | 0.003 | 2.8 | 105 | 2.1 |
| Narmada | 0.004 | 2.8 | 70 | 2.8 |
| North East Coast | 0.003 | 2.5 | 155 | 3.6 |
| Pennar | 0.006 | 1 | 200 | 2.1 |
| Sabarmati | 0.003 | 1 | 155 | 3.6 |
| South Coast | 0.005 | 3.6 | 70 | 2.1 |
| Subarnarekha | 0.002 | 2.8 | 70 | 2.8 |
| Tapi | 0.002 | 2.1 | 155 | 3.6 |
| West Coast | 0.001 | 1 | 70 | 3.6 |
